# Collaboration Between Endocrinologists and Dentists in the Care of Patients with Acromegaly—A Narrative Review

**DOI:** 10.3390/jcm14155511

**Published:** 2025-08-05

**Authors:** Beata Wiśniewska, Kosma Piekarski, Sandra Spychała, Ewelina Golusińska-Kardach, Maria Stelmachowska-Banaś, Marzena Wyganowska

**Affiliations:** 1 Department of Oral Surgery, Periodontology and Oral Mucosa Diseases, Poznan University of Medical Sciences, 10 Fredry Street, 61-701 Poznan, Poland; 2 Arbor Vitae Society of Ethics in Medicine, 103 Marymoncka Street, 01-813 Warsaw, Poland; 3 Department of Endocrinology, The Centre of Postgraduade Medical Education, 99 Marymoncka Street, 01-813 Warsaw, Poland

**Keywords:** acromegaly, growth hormone, oral health, interdisciplinary communication

## Abstract

Acromegaly is caused by an excessive secretion of growth hormone and the secondary elevation of IGF-1 levels, leading to progressive changes in multiple body systems, including the craniofacial region and oral cavity. Dental manifestations such as mandibular overgrowth, macroglossia, malocclusion, periodontal disease, and prosthetic difficulties represent not only a clinical component of the disease but also a significant therapeutic and diagnostic challenge. The aim of this review is to present the current state of knowledge on the relationship between acromegaly and oral health and to analyze the role of interdisciplinary collaboration between endocrinologists and dentists in patient care. For this narrative review, a literature search was conducted in the PubMed, Scopus, and Web of Science databases covering the period from 2000 to 2025. Sixty-two peer-reviewed publications meeting the methodological and thematic criteria were included in the analysis, including original studies, meta-analyses, systematic reviews, and case reports. The results indicate significant correlations between disease activity and the severity of periodontal and microbiological changes, while effective endocrine treatment only results in the partial regression of morphological changes. Particular attention was given to the role of the dentist in recognizing the early symptoms of the disease, planning prosthetic and surgical treatment, and monitoring therapy-related complications. Interdisciplinary collaboration models, including integrated clinics and co-managed care, were also described as optimal systemic solutions for improving treatment quality. The conclusion drawn from the analysis are as follows: there is a need for the permanent integration of dentistry into the standard of interdisciplinary care for patients with acromegaly, in both diagnostic and therapeutic dimensions. Increasing awareness among dentists and developing integrated collaboration models may reduce the time to diagnosis, improve patients’ quality of life, and enable the more effective management of craniofacial complications in the course of this rare disease.

## 1. Introduction

Acromegaly is a rare, chronic endocrine disorder, and in over 95% of cases, it is caused by a pituitary adenoma secreting growth hormone (GH), resulting in the increased production of insulin-like growth factor 1 (IGF-1) in the liver and peripheral tissues [1,2]. Chronic exposure to excess GH and IGF-1 causes the slow but progressive overgrowth of soft tissues and bone structures, including significant changes within the stomatognathic system and craniofacial region, which are often overlooked or treated as separate dental entities [3,4].

In the general population, acromegaly occurs with an estimated prevalence of 50–70 cases per million and an annual incidence of 3–5 cases per million [5]. The average time from the onset of initial symptoms to diagnosis ranges from 6 to 10 years, meaning that patients often remain untreated for a long period while somatic and metabolic changes continue to progress [5,6]. This delay results not only from the low incidence of the disease but also from the nonspecific and multisystemic nature of its symptoms, which are dispersed across various fields of medicine and dentistry [7].

Particular importance is placed on morphological changes within the oral cavity and cranio-maxillofacial structures, which may occur early and be recognized by dentists before a diagnosis is made by an endocrinologist. These symptoms include macroglossia, alveolar process hypertrophy, prognathism, diastemas, occlusal changes, and temporomandibular joint dysfunction [8,9]. In many cases, it is the dentist or orthodontist who first notices abnormalities in the structure and symmetry of the craniofacial region or dentition, which may indicate pituitary disease [10].

Additionally, acromegaly is associated with an increased incidence of periodontal diseases, dental caries, oral infections, and complications following dental and surgical procedures [11,12]. The overgrowth of soft tissues and morphological changes in the skeletal system significantly hinder orthodontic and prosthetic treatment, and, particularly, pose a high risk of failure in implant therapy, especially in the active phase of the disease [13,14]. This requires not only individualized therapeutic approaches but also close interdisciplinary collaboration between dentists, endocrinologists, maxillofacial surgeons, and radiologists.

The existing data indicate that despite numerous oral changes, the topic of collaboration between dentists and endocrinologists remains marginalized in the clinical literature, and care for patients with acromegaly is still mainly conducted in a specialist-monocentric model, with limited access to coordinated dental care [10,15]. Meanwhile, numerous publications emphasize that a multispecialist approach improves not only patients’ quality of life but may also have significant prognostic implications—both in terms of endocrine therapy effectiveness and the preservation of masticatory function, facial esthetics, and psychosocial comfort [16,17].

The aim of this review is a comprehensive analysis of the current literature on the role of dentists in the diagnosis, treatment, and monitoring of patients with acromegaly, and this study discusses the principles and importance of interdisciplinary collaboration between endocrinologists and dental practitioners. In particular, this review addresses the following: dental and craniofacial changes characteristic of acromegaly, challenges associated with dental treatment, the risk of surgical and implant-related complications, and the proposed models of coordinated care.

## 2. Materials and Methods

This study was conducted as a structured narrative literature review aiming to synthesize current scientific knowledge on the relationship between acromegaly and oral health. Particular emphasis was placed on the clinical consequences of hormonal excess within the stomatognathic system, the potential reversibility of craniofacial changes following treatment, the periodontal and microbiological sequelae of chronic endocrine dysregulation, and the role of interdisciplinary care involving dental professionals. The review sought to integrate findings from various domains, including dentistry, endocrinology, oral pathology, radiology, and bone metabolism, in order to develop a comprehensive clinical perspective on this complex patient population.

The literature search was carried out using three major electronic databases: PubMed/MEDLINE, Scopus, and Web of Science. The search strategy included predefined Boolean combinations of MeSH terms and keywords such as “acromegaly” AND “oral health”, “acromegaly” AND “dentistry”, “acromegaly” AND “periodontal disease”, “acromegaly” AND “craniofacial”, “acromegaly” AND “oral microbiota”, “acromegaly” AND “implantology”, and “acromegaly” AND “interdisciplinary care”. Only articles published between January 2000 and May 2025 were included. In addition to database queries, reference lists of key publications were manually screened to identify any relevant sources not captured electronically.

The inclusion criteria encompassed original peer-reviewed clinical studies, systematic reviews, meta-analyses, case reports of significant documentation value, and high-quality narrative reviews that addressed dental, periodontal, microbiological, or anatomical aspects of acromegaly. The exclusion criteria included non-peer-reviewed materials (e.g., editorials, letters to the editor, conference abstracts), duplicate publications, studies unrelated to the stomatognathic system, and reports lacking methodological clarity or clinical relevance.

A total of 62 peer-reviewed publications were ultimately included in the qualitative synthesis. These comprised 20 clinical studies, 5 systematic reviews and meta-analyses, 7 case reports, 23 narrative or expert reviews, and 7 international guidelines or consensus statements. Given the marked heterogeneity in study design, population characteristics, and clinical endpoints, a statistical meta-analysis was not feasible. Instead, a structured narrative synthesis was performed, with studies grouped thematically according to anatomical, therapeutic, microbiological, diagnostic, and interdisciplinary aspects relevant to oral health and systemic manifestations in patients with acromegaly.

The use of a narrative review format was deliberate and enabled the incorporation of findings from diverse medical specialties. This integrative approach allowed for the exploration of clinically significant mechanisms and therapeutic dilemmas that may be overlooked in narrowly focused or quantitatively restricted reviews. The findings are presented in a structured format across thematic sections.

### 2.1. Dental and Craniofacial Changes in the Course of Acromegaly

Prolonged exposure to excess growth hormone (GH) and insulin-like growth factor 1 (IGF-1) leads to characteristic, multidimensional changes within the craniofacial structures and the stomatognathic system. The established phenotype of a patient with acromegaly includes not only mandibular and soft tissue overgrowth but also significant anatomical, functional, and biomechanical transformations in terms of dentition, occlusion, articulation, and upper airway anatomy [1].

Comparative morphological analyses indicate that specific facial soft tissue characteristics may persist even after long-term biochemical remission in acromegaly. Wagenmakers et al. used three-dimensional stereophotogrammetry to assess facial morphology in patients in remission for over two years, revealing increased bizygomatic width, midfacial projection, and facial convexity compared to healthy controls [18]. These findings suggest that certain soft tissue changes may remain irreversible despite hormonal normalization. In contrast, CBCT-based evaluations in patients with active acromegaly demonstrate extensive skeletal alterations, including elongation of the mandibular body, deepening of the gonial angle, and widening of maxillofacial structures, reflecting the progressive nature of bone remodeling during active disease [19].

Several studies have investigated the relationship between the duration of GH/IGF-1 excess and the severity of craniofacial morphological alterations, but the findings remain inconclusive, reflecting the multifactorial nature of disease progression. Some research has demonstrated a positive correlation between the length of disease duration and the degree of mandibular enlargement, soft tissue overgrowth, macroglossia, and dentoalveolar changes. Prolonged exposure to excessive GH/IGF-1 appears to increase the likelihood of irreversible alterations and reduce the probability of morphological regression following biochemical remission [14].

Conversely, recent studies using three-dimensional facial imaging have shown that the severity of certain features—such as nasal width and mandibular elongation—correlates more strongly with current IGF-1 levels, while the association with disease duration did not reach statistical significance [20]. Furthermore, postoperative analyses have indicated that the extent of facial soft tissue regression, particularly nasal narrowing, was significantly associated with reductions in GH and IGF-1 levels, independently of the duration of the disease [14].

These findings suggest that the impact of disease duration may not be linear and that the severity of craniofacial changes is influenced not only by time but also by the intensity of hormonal exposure, tissue sensitivity, and the timing of diagnosis. It is generally accepted that soft tissue changes can develop relatively rapidly, whereas osseous alterations—such as mandibular body elongation or mandibular angle deepening—progress more slowly but tend to persist, often resisting reversal. This multifactorial model of morphological progression is increasingly supported in the literature and underscores the clinical importance of early diagnosis and interdisciplinary treatment planning.

The most frequently described skeletal alteration is mandibular prognathism, resulting from disproportionate growth of the lower facial arch, accompanied by relatively less maxillary enlargement. Imaging studies reveal elongation of the mandibular body and ramus, deepening of the mandibular angle, and condylar hypertrophy [4]. Cephalometric studies have shown that patients with active acromegaly exhibit significant elongation of the mandibular body and ramus, widening of the cranial base, and deepening of the temporal fossae compared to healthy controls [21]. Mandibular overgrowth disrupts the relationship between dental arches, leading to anterior open bite, crossbite, and tooth displacements, most commonly in the form of characteristic diastemas [9]. In a cephalometric study by Balos Tuncer et al., significant alterations in mandibular angle and basal bone length were observed in patients with acromegaly compared to healthy controls, with a high prevalence of occlusal abnormalities [22]. Importantly, even in patients in biochemical remission, at least one morphological mandibular feature remained persistent.

Another significant clinical manifestation is macroglossia, resulting from the hypertrophy of tongue muscles, increased connective tissue, and vascularity. Tongue enlargement may lead to phonation disorders, swallowing difficulties, and mechanical pressure on the dental arches, exacerbating existing occlusal abnormalities [23]. MRI-based measurements have confirmed that patients with acromegaly exhibit significantly enlarged tongue volumes and changes in muscle structure compared to healthy controls, as demonstrated in a clinical series using TrueFISP imaging [24].

Another typical finding is gingival hypertrophy, which often masks early signs of periodontal disease. Excess IGF-1 induces fibroblast and vascular proliferation, which results in the overgrowth of oral soft tissues, thereby hindering proper hygiene and predisposing the patient to periodontopathies. Therapeutic interventions in acromegaly, particularly the administration of somatostatin analogs (SSAs), may influence the severity of gingival overgrowth and periodontal inflammation. There are case-based observations suggesting that effective endocrine treatment leading to the biochemical control of the disease can reduce soft tissue hypertrophy in the oral cavity and improve periodontal parameters, including decreased inflammatory indices and enhanced tolerance to hygiene procedures. In a clinical study by Capoglu et al., gingival hypertrophy was identified in a subset of patients and showed a tendency to improve after biochemical control of the disease was achieved [25].

However, these findings remain limited to individual reports, and the overall effect of hormonal therapy on structural periodontal changes has not been thoroughly investigated. Longitudinal studies are needed to assess whether these improvements are sustained or merely transient and closely tied to disease activity. In a comparative study carried out by Harb et al., which involved patients with acromegaly and healthy controls, significantly increased gingival inflammation and clinical attachment loss were reported in the acromegaly group, highlighting the impact of the disease on periodontal health [11].

Changes in the temporomandibular joint (TMJ) in patients with acromegaly result from functional overload and hypertrophy of the mandibular condyle. Cone beam computed tomography (CBCT) studies have demonstrated significant thickening of the mandibular head, enlargement of the condylar process, and a reduction in the height of the articular fossa, and these phenomena are often accompanied by secondary degenerative changes of the articular disk [19]. Clinical symptoms include limited mouth opening, deviation of the mandibular trajectory, joint crepitus, and chronic pain [26].

The anatomical alterations of the temporomandibular joint and mandible observed in acromegaly may also impact masticatory muscle function and occlusal stability. Although few studies have directly assessed this relationship in acromegalic patients, electromyographic (EMG) findings from related conditions—such as pain-related temporomandibular disorders—indicate that structural and functional imbalance may lead to increased baseline muscle tone and altered activation patterns in the masseter and temporalis muscles. While direct evidence from acromegalic patients is limited, EMG studies in related conditions—such as pain-related temporomandibular disorders—have demonstrated asymmetrical activity and altered function of the masticatory muscles, supporting the hypothesis that structural imbalance may lead to neuromuscular adaptation and functional overload [27]. Furthermore, clinical observations point to decreased bite force and impaired masticatory performance, particularly in advanced cases with pronounced prognathism and diastemas.

In women with acromegaly, hormonal factors may additionally modulate temporomandibular joint (TMJ) pain perception. Estrogen plays a complex regulatory role in TMJ function, influencing inflammatory pathways, nociceptive signaling, and joint tissue remodeling. The disruption of estrogen homeostasis—frequently observed in pituitary disorders such as acromegaly—may therefore contribute to altered pain thresholds and increased vulnerability to TMJ dysfunction. Recent systematic reviews have highlighted the significance of sex hormones, particularly estrogens, in modulating pain perception and joint pathology in temporomandibular disorders, underscoring the importance of considering endocrine status in female patients presenting with TMJ-related symptoms [28].

Emerging evidence also suggests that the prevalence and severity of stomatognathic alterations in acromegaly may differ by sex and age at diagnosis. In a recent cross-sectional study involving acromegalic patients of both sexes, female patients more frequently reported functional disturbances such as temporomandibular joint pain, open bite, and macroglossia, while also exhibiting a higher incidence of occlusal irregularities and difficulties with mastication. These findings may reflect a combination of anatomical, hormonal, and pain modulation factors that are sex-specific, particularly in light of the known interactions between estrogen and TMJ nociception [4]. Furthermore, age at diagnosis appears to influence the extent and irreversibility of skeletal changes. Patients diagnosed at a younger age exhibited more pronounced craniofacial alterations, including mandibular overgrowth and dental spacing, suggesting that earlier onset of hormonal excess allows for a longer period of progressive structural remodeling [4]. These observations underscore the importance of timely diagnosis and sex-specific considerations in the clinical evaluation of craniofacial symptoms in acromegaly.

Craniofacial bone changes also include the thickening of the frontal and zygomatic bones, enlargement of the paranasal sinuses, and elongation of the nose and broadening of its base, giving the face a characteristic “coarse” appearance. Radiological studies in patients with acromegaly have demonstrated significantly greater pneumatization of the frontal sinuses and increased maxillary bone and hard palate volume [7].

The functional consequences of anatomical changes are also significant. The hypertrophy of the tongue, soft palate tissues, and posterior pharyngeal wall leads to narrowing of the retropalatal space and the development of obstructive sleep apnea (OSA). According to a systematic review and meta-analysis carried out by Parolin et al., the prevalence of OSA in patients with acromegaly ranges from 60% to over 80%, with macroglossia and pharyngeal soft tissue hypertrophy considered major contributing factors [29]. OSA additionally increases cardiovascular risk and requires active interdisciplinary treatment, often involving a maxillofacial surgeon and an otolaryngologist.

Therapeutic difficulties in the oral and craniofacial region not only result from altered anatomy but also from the impact of GH/IGF-1 on bone metabolism. Data suggest that patients with active acromegaly may have an imbalance between bone resorption and formation, which translates into poorer implant and surgical treatment outcomes. Hormonal dysregulation, including excess GH and IGF-1, has been shown to negatively affect osseointegration and bone remodeling, increasing the risk of marginal bone loss and implant failure, particularly in patients without biochemical disease control [30].

The final clinical picture of dental and craniofacial changes in acromegaly is complex, dynamic, and varies in severity. Their presence may not only serve as a diagnostic aid but also as a major therapeutic challenge. Including them in the routine assessment of patients—carried out by dentists and in close collaboration with an endocrinologist—is essential for effective, integrated care.

From a clinical standpoint, dental practitioners should be aware of the specific challenges associated with treating patients diagnosed with acromegaly and adapt their management protocols accordingly. Given the increased incidence of gingival hypertrophy, periodontal inflammation, and oral soft tissue overgrowth, these individuals should undergo dental check-ups at more frequent intervals—ideally every 3 to 6 months—to allow for early detection and treatment of periodontal pathology [18]. Furthermore, occlusal instability, macroglossia, and temporomandibular joint dysfunction require individualized occlusal assessments and may necessitate splint therapy or periodic occlusal equilibration to minimize discomfort and functional overload [4]. For restorative procedures, clinicians are advised to select high-strength, wear-resistant materials that are better suited to withstand increased masticatory forces and bruxism-like patterns commonly reported in this population. Implant placement should be postponed until biochemical remission is achieved, given the elevated risk of marginal bone loss and implant failure in the context of active disease and disturbed bone remodeling dynamics [30]. Above all, interdisciplinary coordination with endocrinologists and maxillofacial specialists remains essential to ensure comprehensive and safe long-term care.

### 2.2. Impact of Endocrinological Treatment on Oral Health

The treatment of acromegaly, regardless of the therapeutic strategy employed, significantly affects the morphology, function, and biological condition of oral tissues and craniofacial structures. An effective reduction in GH and IGF-1 levels results in stabilization or the partial regression of hypertrophic changes but does not always result in the full anatomical and functional parameters normalizations of the stomatognathic system [31]. It should be emphasized that the choice of treatment modality—surgical, pharmacological, or radiotherapeutic—and the degree of hormonal control achieved critically influence the extent, rate, and potential reversibility of skeletal and soft tissue alterations associated with acromegaly [32].

Transsphenoidal adenomectomy remains the first-line treatment for acromegaly and is associated with favorable outcomes in terms of disease control and the stabilization of craniofacial abnormalities. Achieving biochemical remission—typically defined by the normalization of IGF-1 and the suppression of GH—can halt the progression of soft tissue hypertrophy and, in some cases, induce partial regression, particularly in the tongue, lips, and facial musculature [31]. However, osseous changes affecting the mandible, maxilla, and cranial base tend to be irreversible, given their progressive remodeling over years of hormonal excess. In a study carried out by Wagenmakers et al., three-dimensional facial analysis in patients in long-term remission revealed persistent mandibular overgrowth and maxillary expansion, despite successful endocrine therapy [18]. This underscores the need for early diagnosis and, in selected cases, the consideration of adjunctive orthognathic or reconstructive interventions to address residual skeletal deformities.

The reversibility of craniofacial and oral changes in patients with acromegaly is influenced by several clinical and demographic factors. Age at diagnosis appears to be a key determinant, as younger patients demonstrate greater potential for soft tissue remodeling and partial skeletal adaptation following biochemical remission. In contrast, patients diagnosed after the fifth decade of life more often present with fixed osseous deformities, likely due to longer exposure and the reduced regenerative capacity of bone tissue [33]. Sex-based differences have also been reported, with women showing a higher prevalence of persistent stomatognathic abnormalities, including gingival overgrowth and occlusal instability, potentially related to hormonal modulation and temporomandibular joint sensitivity [34]. Tumor size and invasiveness play a significant role, as macroadenomas tend to be associated with higher GH/IGF-1 output and longer disease duration, both of which correlate with more severe craniofacial involvement and limited post-treatment regression [35]. Notably, the overall duration of active disease prior to treatment initiation remains one of the most consistent predictors of the extent and permanence of morphological changes in the oral cavity, mandible, and midface skeleton [36]. The early diagnosis and timely initiation of endocrine therapy therefore remain crucial for minimizing long-term structural alterations and improving functional oral outcomes. A structured summary of impact of endocrinological treatment on oral health in acromegaly is presented in Table 1.

Long-term clinical observations following pituitary adenomectomy indicate that, despite biochemical remission and the normalization of IGF-1 levels, the regression of craniofacial and oral morphological changes is often incomplete and varies significantly between individuals [37]. Notably, in a cohort analyzed by Wagenmakers et al., patients in long-term remission showed persistent mandibular overgrowth and maxillary expansion despite successful endocrine therapy, as demonstrated through three-dimensional facial analysis [18].

These findings suggest that even long-term endocrine control does not necessarily reverse established bony deformities, which has important implications for dental, prosthetic, and surgical planning in patients with acromegaly.

Pharmacotherapy is the cornerstone of treatment in cases when surgery is unsuccessful, contraindicated, or when hormonal control is needed prior to planned procedures. The most commonly used drugs include somatostatin analogs (SSAs), dopamine agonists, and the GH receptor antagonist pegvisomant [38]. These medications not only influence GH and IGF-1 levels but also exert downstream effects on bone metabolism, oral mucosal perfusion, and fibroblast activity. Studies indicate that well-controlled acromegaly may attenuate periodontal inflammation and soft tissue overgrowth [39]. Moreover, a reduction in bleeding index (BI) and plaque index (PI) has been observed in patients undergoing pharmacological treatment, suggesting a favorable effect of hormonal control on periodontal conditions. Pegvisomant, although it does not reduce GH levels directly, effectively decreases IGF-1 and is associated with improved soft tissue status and quality of life. In a 2022 retrospective-prospective study involving 28 patients treated with pegvisomant, 71% reported improved prosthetic comfort within 9 months of treatment initiation [40]. While evidence regarding its direct oral effects is limited, clinical observations suggest potential benefits in masticatory function and articulation due to soft tissue remodeling [41]. Interdisciplinary monitoring remains essential, particularly in patients undergoing long-term pharmacotherapy or requiring dental prosthetic planning.

Pituitary radiotherapy, although less commonly used in the treatment of acromegaly, carries a substantial risk of inducing secondary hypopituitarism. The resultant hormonal deficiencies, particularly growth hormone and gonadal axis suppression, have been associated with long-term metabolic disturbances, including reduced bone mineral density. These changes may lead to weakening of the mandibular bone and an increased risk of tooth loss in affected individuals [42]. Given the potential skeletal implications, dental interventions—especially those involving implants or prosthetics—should be carefully planned in collaboration with the endocrinologist and performed after the stabilization of pituitary function.

Available studies investigating the impact of endocrinological treatment on oral health in acromegaly have demonstrated a consistent trend toward improvement in soft tissue parameters, particularly in patients achieving long-term biochemical remission. However, methodological limitations in many reports—such as small sample sizes, short follow-up periods, and lack of stratification by disease severity—impair definitive conclusions regarding the magnitude and reversibility of oral manifestations. Regarding the effect of treatment on hard tissues, the available evidence suggests that endocrinological therapy primarily affects soft tissue components, such as gingiva, tongue, and oropharyngeal musculature. Partial functional normalization has been described, including improved articulation and prosthetic tolerance; however, structural changes in osseous tissues—particularly the mandible, alveolar bone, and skull base—tend to persist despite long-term biochemical remission [35]. No high-quality studies to date have demonstrated the consistent regeneration or remodeling of alveolar bone following SSA or pegvisomant treatment. This underscores the importance of early diagnosis and timely surgical or pharmacological intervention before permanent skeletal deformities are established.

In clinical practice, the feasibility and prognosis of implant treatment in patients with acromegaly must take into account alterations in maxillary and mandibular bone quality. Long-standing GH/IGF-1 excess has been shown to disrupt bone remodeling and may lead to reduced bone density, particularly in the alveolar process. As highlighted by Giustina (2023), acromegaly is associated with site-specific skeletal changes that can compromise implant integration and increase the risk of peri-implant complications [43]. Therefore, preoperative assessment should include the evaluation of IGF-1 levels, periodontal condition, and occlusal stability. Despite these challenges, implant therapy may still be effective if coordinated within an interdisciplinary treatment plan and initiated after biochemical control of the disease has been achieved.

Given the multisystemic nature of acromegaly and its complex oral manifestations, an interdisciplinary model of care is strongly recommended. Optimal treatment planning should involve close cooperation between endocrinologists, oral and maxillofacial surgeons, orthodontists, and periodontists. Endocrine stabilization should always precede elective dental and surgical procedures, and therapeutic decisions should consider both systemic and local factors. The attending dentist should be fully informed of the patient’s hormonal status, medication history, and comorbidities that may influence tissue healing and implant success.

Routine dental monitoring in patients with acromegaly should include the periodic assessment of occlusal relationships (e.g., progression of anterior open bite or crossbite), periodontal status (with attention to inflammation and attachment loss), prosthetic fit and comfort, and documentation of soft tissue hypertrophy, particularly gingival overgrowth and macroglossia. These assessments should be conducted at regular intervals—preferably every 6 months—and adapted to the disease control stage and the patient’s treatment history.

The conclusions from current research clearly indicate that the effective endocrinological treatment of acromegaly has a positive impact on oral health, reduces the risk of periodontal complications, and improves patient’s quality of life. Nevertheless, the full normalization of the anatomical and functional conditions of the stomatognathic system is not achievable in all patients, which justifies the necessity for individualized, multidisciplinary dental-endocrinological care.

### 2.3. Dental and Implant Treatment in Patients with Acromegaly

Dental treatment in patients with acromegaly poses a significant clinical challenge due to complex morphological, functional, and metabolic changes within the stomatognathic system. Progressive craniofacial bone overgrowth, soft tissue hypertrophy, occlusal alterations, and distorted dental arch anatomy necessitate an individualized, multidisciplinary approach. Particular attention must be focused on the planning of prosthetic and implant therapy, which should be preceded by an evaluation of hormonal status, bone metabolism, and coordination with the attending endocrinologist.

During the active phase of the disease, which is characterized by elevated levels of GH and IGF-1, elective oral procedures should generally be postponed, as active acromegaly is associated with impaired bone remodeling and metabolic instability. A meta-analysis by Mazziotti et al. confirmed that uncontrolled disease increases the risk of skeletal fragility and abnormal bone turnover, which may compromise osseointegration and wound healing [44].

Anatomical changes in the jaws, including mandibular prognathism and maxillary narrowing, often result in occlusal disharmony, loss of interarch stability, and functional edentulism despite the presence of natural teeth. In addition, tooth mobility as a result of periodontal disease may hinder the achievement of a stable centric occlusion, which is a prerequisite for successful prosthetic rehabilitation [45]. In many cases, conventional removable dentures are poorly tolerated or ineffective. In carefully selected patients with stable biochemical disease and favorable bone conditions, implant-supported prostheses may offer a more reliable alternative [45].

Implant treatment in patients with acromegaly requires individualized planning due to complex alterations in bone remodeling, immune responses, and anatomical structures. Elevated levels of GH and IGF-1 influence both osteoblast and osteoclast activity, which may compromise peri-implant bone stability and long-term osseointegration [43]. A clinical report by Kernen et al. highlighted that although implants can be successfully integrated in patients with controlled acromegaly, the incidence of peri-implant complications such as marginal bone loss and soft tissue inflammation remains significantly elevated compared to that in healthy individuals [45]. Consequently, implant placement should be restricted to patients in confirmed biochemical remission and preceded by comprehensive diagnostic assessments, including CBCT imaging, periodontal evaluation, and screening for metabolic comorbidities. Adjunctive supportive strategies—such as probiotic supplementation, the optimization of vitamin D and calcium levels, and glycemic control—are recommended to enhance treatment outcomes and reduce risk.

In cases of severe craniofacial deformities or functional occlusal disturbances, orthognathic surgery may be considered. However, these procedures should be delayed until the patient achieves full hormonal stabilization and must be planned in coordination with an interdisciplinary team including an endocrinologist, orthodontist, and maxillofacial surgeon [46]. Although not yet validated specifically in acromegalic patients, recent advances in 3D surgical planning and patient-specific implants (PSIs) have opened new opportunities for personalized craniofacial reconstruction in cases of severe skeletal deformities [47]. A structured summary of clinical considerations and recommendations for implant and prosthetic treatment in acromegaly is presented in Table 2.

### 2.4. Periodontal Diseases and Oral Microbiota in Acromegaly

As a chronic endocrine disorder, acromegaly significantly alters the immunological and inflammatory profile of oral tissues, contributing to an increased susceptibility to periodontal disease. Inflammatory dysregulation associated with excess GH and IGF-1 affects both innate and adaptive immune mechanisms, promoting tissue hypertrophy, prolonged neutrophil activity, and the increased expression of proinflammatory cytokines, including IL-1β, IL-6, and TNF-α. These molecular alterations facilitate connective tissue breakdown and alveolar bone resorption. Clinical evidence from patients with acromegaly supports this mechanism, showing increased periodontal inflammation and elevated IL 6 levels even in biochemically stable individuals [48].

Emerging evidence suggests that periodontal health in patients with acromegaly may both reflect and influence the systemic endocrine environment. Inflammatory cytokines such as IL-6 and TNF-α—which are frequently elevated in chronic periodontitis—can modulate GH receptor signaling and IGF-1 bioavailability, potentially contributing to variable therapeutic responses. Although causality has not been established, studies have highlighted a possible bidirectional interaction between biochemical disease activity and periodontal inflammation [48].

This interplay between systemic hormonal dysregulation and oral inflammation underscores the importance of a coordinated therapeutic approach. Although somatostatin analogs may exert indirect anti-inflammatory effects by lowering systemic GH and IGF-1 levels, current evidence suggests that their impact on periodontal status is modest unless accompanied by effective local therapy. A study carried out by Jain et al. demonstrated altered cytokine profiles in patients with acromegaly, especially in those with coexisting metabolic disturbances, suggesting a potential feedback mechanism between periodontal inflammation and endocrine activity [48]. Therefore, the integration of periodontal care into the routine management of acromegalic patients may offer systemic benefits beyond oral health alone.

The pharmacological treatment of acromegaly may indirectly affect salivary composition and the oral environment. Although somatostatin analogs can reduce salivary flow and alter mucosal function, emerging evidence suggests that qualitative changes in saliva play a more critical role in oral microbial imbalance. Analyses of saliva samples from patients with acromegaly have shown the decreased activity of key antimicrobial enzymes such as salivary peroxidase and α-amylase, and lowered concentrations of bicarbonate ions. This impaired buffering capacity favors acidogenic and proteolytic bacterial species and reduces the natural antimicrobial defense of the oral environment. Notably, these biochemical alterations may persist even in the absence of subjective xerostomia, further complicating long-term microbial homeostasis [49].

Another risk factor for the progression of periodontal disease is the difficulty in maintaining oral hygiene due to tooth displacement, alveolar process hypertrophy, and macroglossia. These changes hinder effective tooth brushing, which may lead to excessive plaque and calculus accumulation. In many patients, effective plaque control is further compromised by limited mandibular mobility and TMJ-related pain, which reduce the jaw’s opening range and impede access to posterior teeth. Studies using CBCT imaging have shown structural joint alterations—including condylar thickening and articular disk displacement—that correlate with clinical findings such as jaw deviation and muscular tension. These functional limitations, when combined with gingival overgrowth and altered arch morphology, may significantly impair the feasibility of standard oral hygiene techniques [19]. It is therefore recommended that patients with acromegaly be enrolled in individualized periodontal prevention programs, which should include more frequent follow-up visits, professional dental cleanings, and the use of oral probiotics. These probiotics have been shown to help stabilize the oral microbiota and reduce inflammation in soft tissues [50]. A structured summary of determinants of periodontal disease and microbial imbalance in acromegaly is presented in Table 3.

In light of the multifactorial risks affecting periodontal health in acromegaly, individualized prevention strategies should be considered an integral part of dental management. Periodontal follow-up is generally recommended every 3 to 4 months, with professional supragingival and subgingival debridement tailored to clinical status. Instruction in the use of modified interdental brushes or water flossers may benefit patients with malpositioned teeth or macroglossia. Additionally, the use of oral probiotics containing Lactobacillus reuteri or Streptococcus salivarius has been shown to reduce periodontal inflammation and stabilize the dysbiotic microbiome in endocrine-related conditions. Alcohol-free and chlorhexidine-free antiseptic rinses may be preferred to preserve microbiome diversity and minimize mucosal irritation [51].

### 2.5. The Role of the Dentist in the Diagnosis of Acromegaly

In the context of the delayed diagnosis of acromegaly—which, according to epidemiological data, averages between 6 and 10 years from the onset of the first clinical symptoms—the role of the dentist in the diagnostic process becomes particularly significant. Changes occurring in the craniofacial region, oral cavity, and masticatory system are among the earliest and often the first visible manifestations of the disease, creating an opportunity for their detection at a stage when systemic symptoms are still nonspecific or mild. Regular dental check-ups, the widespread availability of dental services, and patients’ tendency to seek dental consultation for “aesthetic” or “mechanical” concerns before visiting a primary care physician position the dentist as a potential first link in the diagnostic chain leading to the recognition of acromegaly.

Recent analyses from large international databases indicate that the average diagnostic delay in acromegaly remains substantial, typically ranging from 5 to over 10 years depending on the geographical location, the healthcare system’s efficiency, and the patient’s access to specialized endocrinological services [36]. This delay is often attributed to the insidious onset of symptoms and the initial presentation of non-specific complaints. Misdiagnoses in the early stages are common, with patients frequently being treated for temporomandibular joint dysfunction, bruxism, or evolving malocclusion without adequate systemic assessment [5]. Notably, dental practitioners may be the first among healthcare providers to encounter early oro-facial manifestations of acromegaly. Questionnaire-based studies have shown that while features such as diastemas, macroglossia, or facial disproportion are often recognized during routine dental evaluations, the suspicion of systemic endocrine disease is rarely raised. Only a minority of dental professionals report ever having referred a patient for further endocrinological assessment [12]. These findings highlight the strategic role of dentists in the early identification of acromegaly and underscore the importance of targeted educational initiatives aimed at improving diagnostic vigilance within the dental community.

Early-stage manifestations of acromegaly within the stomatognathic system are often subtle yet pathognomonic when assessed in conjunction. Among dental findings, progressive diastemas and occlusal changes in adult patients without prior orthodontic treatment may represent the earliest visible signs. These are frequently accompanied by incipient mandibular prognathism and lower jaw asymmetry, which can be verified through photographic or cephalometric comparisons. In the domain of soft tissues, macroglossia and mild gingival overgrowth may appear, altering phonation, swallowing, and prosthesis stability. Initial temporomandibular joint (TMJ) symptoms typically include joint clicking and deviations during mandibular opening, and they are often misinterpreted as parafunctional disorders or age-related changes.

In later stages of acromegaly, patients frequently exhibit functional edentulism and malocclusion that may resist prosthetic or orthodontic correction due to significant mandibular overgrowth and altered skeletal relationships. Cephalometric and craniofacial studies have documented increased facial height, mandibular prognathism, and a larger gonial angle in affected patients [22]. Furthermore, a comprehensive review has reported that approximately 40–43% of acromegaly patients present with dental diastemas, 22–24% present with mandibular overgrowth, 20–22% present with mandibular prognathism, and 54–58% present with macroglossia, all of which can exacerbate occlusal disharmony and compromise dental rehabilitation [4].

Classifying these manifestations by anatomical region—dentition, mandible, soft tissues, and facial profile—and recognizing their progression from early to late stages enhances clinical awareness and supports the earlier suspicion of systemic disease. This anatomical framework is particularly applicable in general dental, prosthodontic, and orthodontic practice, where such changes may be observed long before an endocrinological diagnosis is made.

Symptoms that should raise the dentist’s suspicion include progressive diastemas, occlusal changes in adult patients without a prior orthodontic history, mandibular and chin enlargement, speech articulation difficulties, problems with prosthetic rehabilitation, and decreasing tolerance of fixed or removable dentures without a clear cause. Additionally, macroglossia, soft tissue overgrowth in the oral cavity, worsening malocclusions, and recurrent temporomandibular joint pain are often early manifestations of acromegaly and may be misinterpreted as periodontal issues, bruxism, or muscular parafunctions.

According to a comprehensive literature review, dental diastemas were reported in 40–43% of acromegaly cases, mandibular overgrowth in 22–24%, mandibular prognathism in 20–22%, and macroglossia in 54–58% [4]. Moreover, in a questionnaire-based study of 426 dentists and orthodontists, while most practitioners noted mandibular prognathism and tongue enlargement, only about 11.3% had ever referred a patient with suspected acromegaly for endocrine evaluation [12].

In addition to skeletal and occlusal abnormalities, acromegaly frequently involves alterations of the oral soft tissues and mucosa, which may further aid early recognition by dental professionals. Gingival overgrowth is among the most common findings and may be mistaken for medication-induced hyperplasia or chronic inflammatory conditions. However, in acromegaly, this type of hypertrophy occurs independently of pharmacologic stimuli and is driven by the proliferative effect of IGF-1 on fibroblasts and microvasculature, contributing to pseudopocket formation and the masking of periodontal inflammation [25].

Macroglossia, while already noted as a structural symptom, also manifests with secondary mucosal changes, including tongue scalloping, lateral ulcerations due to trauma, and impaired phonation. In some patients, repeated episodes of mucosal irritation lead to chronic erythema or keratosis of the dorsal tongue surface. These alterations are likely associated with hypertrophy-driven ischemia and compromised microcirculation. Cortet-Rudelli emphasized the diagnostic importance of oral manifestations in acromegaly, particularly macroglossia, gingival overgrowth, mucosal thickening, and speech difficulties, which may precede other systemic symptoms [52]. Historical histopathological data also confirmed substantial soft tissue overgrowth and structural tongue changes in acromegalic patients, independent of thyroid dysfunction [53].

The buccal and labial mucosae in patients with acromegaly often appear thickened and less pliable, sometimes compromising denture retention and altering speech articulation. In prosthesis wearers, these mucosal changes can mimic allergic or mechanical issues, yet persist despite adjustments. Case reports, such as a full-mouth prosthetic rehabilitation example, describe persistent occlusal instability and the need for customized devices due to mucosal alterations [4]. In one case, a lip-bumper prosthesis was specifically utilized to accommodate musculo-mucosal changes in an acromegalic patient. The combination of gingival hypertrophy, macroglossia with trauma-related lesions, and ongoing prosthetic intolerance should prompt suspicion of systemic proliferative disease, particularly when they occur together [4,9,53,54].

The diagnostic importance of the dentist is also underscored by population-based data. In a questionnaire-based study by Preo et al., conducted among 426 Italian dentists and orthodontists, 10.8% reported having previously treated a patient with acromegaly, and 11.3% stated that they had referred at least one patient for endocrine evaluation due to oro-facial abnormalities. These findings suggest that the potential effectiveness of the early dental detection of acromegaly may reach up to 2–3% among patients presenting with nonspecific occlusal or esthetic symptoms, particularly when assessed by experienced clinicians in prosthodontic or orthodontic settings [12].

Despite the presence of characteristic oral and craniofacial features, acromegaly is frequently misinterpreted in dental practice, especially in its early stages. The most common misdiagnoses include bruxism, temporomandibular disorders (TMDs), idiopathic mandibular asymmetry, or the relapse of previously treated malocclusion. These diagnostic confusions arise due to the gradual nature of morphological changes and their overlap with prevalent non-endocrine dental conditions [12].

In a multicenter observational study conducted in Germany, 38% of patients with confirmed acromegaly had previously received at least one dental diagnosis unrelated to endocrine pathology—most often bruxism or TMD—resulting in delayed referral for endocrinological evaluation [4]. A literature review by De Stefani et al. similarly emphasizes that dental professionals are often among the first to observe changes in facial or occlusal morphology, but these are frequently overlooked or misattributed [4].

This pattern highlights the diagnostic vulnerability at the interface of dentistry and systemic medicine. Because mandibular enlargement, joint discomfort, or bite changes often progress insidiously, they are prone to under-recognition, especially in adult patients without a clear triggering event. Moreover, routine dental evaluations rarely include a longitudinal comparison of craniofacial morphology or the consideration of systemic hormonal influence. As a result, patients may receive occlusal splints, physiotherapy, or prosthetic adjustments for symptoms that are, in fact, manifestations of an evolving endocrinopathy.

Integrating clinical red flags into routine dental reasoning—such as rapid diastema formation, prosthetic instability despite good fit, persistent TMJ dysfunction resistant to conventional therapy, or concurrent soft tissue overgrowth—may help mitigate this problem. Education in pattern recognition and referral algorithms is essential to reduce the frequency of missed opportunities for early diagnosis.

Artificial intelligence tools are becoming increasingly relevant for differential diagnosis, especially in the analysis of facial morphological changes typical of acromegaly. In the study carried out by Ryu et al., deep learning algorithms achieved a sensitivity of 96.3% and a specificity of 94.8% in identifying acromegalic facial phenotypes based solely on frontal facial photographs. Although these tools are not yet commonly used in dental practice, they may become valuable aids in identifying patients who require further endocrinological evaluation in the future [4].

The clinical interface between dentistry and systemic medicine has become increasingly relevant in recent years, particularly in the context of chronic diseases with oral manifestations such as diabetes, autoimmune disorders, and acromegaly. While undergraduate and postgraduate dental education varies considerably across countries and institutions, several expert groups have drawn attention to the potential value of expanding interdisciplinary diagnostic competencies. Recognizing the morphological and mucosal indicators of systemic disease—such as disproportionate mandibular growth, macroglossia, or unexplained prosthetic instability—could be considered a component of clinical vigilance rather than a formal diagnostic obligation [12].

International organizations, including the American Dental Education Association (ADEA) and the European Federation of Periodontology (EFP), have proposed the broader inclusion of medical modules in dental training, with particular emphasis on the early identification of systemic signs in oral tissues and appropriate referral protocols [25]. Such educational frameworks do not aim to transform dentists into diagnosticians of endocrine pathology but rather to enhance communication between disciplines and reduce diagnostic delay in complex or rare disorders.

In the context of acromegaly, familiarity with typical phenotypic changes and awareness of their potential systemic basis may support more timely and coordinated care. While the implementation of standardized guidelines remains limited, practical exposure to clinical case studies and interdisciplinary consultation models may help bridge the current knowledge gap [15].

Findings from the literature and clinical experience clearly indicate that dentists occupy a unique position to observe the earliest changes suggestive of acromegaly. However, the effective utilization of this diagnostic potential requires appropriate awareness of the symptoms, clinical vigilance, and a willingness to cooperate with other medical specialists. Including the topic of endocrine diseases in postgraduate and specialty dental education could significantly improve the quality of interdisciplinary care and reduce the time to diagnosis in this diagnostically challenging condition.

Although a definitive diagnosis of acromegaly requires biochemical testing and pituitary gland imaging, dentists may observe oral and craniofacial features that warrant interdisciplinary consideration. In daily practice, certain clinical constellations may be suggestive of underlying endocrine dysregulation, particularly when they evolve gradually and do not respond to standard dental management.

For instance, adult patients with progressive mandibular enlargement or anterior facial protrusion—unrelated to trauma, orthodontic relapse, or neoplastic disease—may benefit from broader diagnostic reflection. Similarly, individuals reporting frequent need for prosthetic adjustments due to perceived denture instability, despite optimal technical fit, might present with early manifestations of soft tissue hypertrophy or occlusal drift [53]. The presence of macroglossia accompanied by scalloping, persistent TMJ dysfunction unresponsive to splint therapy, or unexplained diastema formation may also raise concern when occurring in combination [4].

While these findings are not pathognomonic, they may support a cautious referral to an endocrinologist—especially when confirmed by comparative records or when accompanied by systemic complaints such as changes in hand or shoe size, fatigue, or voice deepening. Including a short summary of dental findings in the referral documentation may enhance interprofessional communication and streamline the diagnostic process [12,54].

### 2.6. Models of Interdisciplinary Collaboration in the Care of Patients with Acromegaly

As a chronic, multisystemic condition, acromegaly requires not only precise endocrinological treatment but also integrated specialist care, involving disciplines such as dentistry, maxillofacial surgery, otolaryngology, neurology, cardiology, ophthalmology, and clinical psychology. The involvement of these specialties is clinically justified by the broad spectrum of systemic and local complications associated with acromegaly [55]. For example, otolaryngologists play a crucial role in the evaluation and management of obstructive sleep apnea, which affects up to 70% of patients due to soft tissue hypertrophy and upper airway narrowing [35]. Neurological consultation may be required in cases of cranial nerve compression, sensory deficits, or headaches related to tumor expansion. Clinical psychologists are essential in supporting patients with body image disturbances, depression, or treatment-related distress [56]. Cardiology input is frequently needed for hypertension, arrhythmias, or cardiomyopathy, which may coexist with oral complications such as OSA-related bruxism or xerogenic medication side effects [57]. Due to the heterogeneous course of the disease and the often subtle initial symptoms, the effective management of patients with acromegaly necessitates close, bidirectional collaboration between endocrinologists and dentists—not only in the context of detecting signs of the disease but also in the long-term therapeutic planning and monitoring of complications. In practical terms, this bidirectional model assumes more than occasional consultation. It involves structured communication pathways whereby dentists can refer patients for endocrine evaluation upon observing progressive craniofacial changes [12], while endocrinologists can routinely include dental assessments in treatment protocols, particularly before surgical or pharmacologic interventions [4]. This model also encourages joint reviews of diagnostic imaging (e.g., CBCT, MRI), co-authored treatment plans for high-risk patients (e.g., those with severe malocclusion, macroglossia, or implant complications), and shared follow-up strategies for monitoring therapy-related effects. Recent studies have also shown that machine learning can support the early recognition of acromegaly through automated detection of facial changes, emphasizing the need for interdisciplinary vigilance [58].

According to current Endocrine Society Guidelines, care for patients with acromegaly should be delivered within a multidisciplinary care model, involving regular information exchange between specialists and shared decision-making in therapy planning [15]. These recommendations are outlined in the 2014 guideline [10], which highlights the importance of evaluating craniofacial abnormalities, oral symptoms, and conditions such as obstructive sleep apnea as part of the baseline assessment. It also supports the involvement of dental professionals as part of the multidisciplinary team [10].

From a practical standpoint, the most effective model of collaboration is co-managed care, in which the endocrinologist is responsible for hormonal diagnostics, causal treatment, and the monitoring of biochemical remission, while the dentist plays both a diagnostic role (e.g., identifying craniofacial changes and periodontal disease) and a therapeutic role—implementing prosthodontic, periodontal, or surgical treatments. Therapeutic procedures performed by dental professionals in this context may include non-surgical periodontal therapy (e.g., scaling and root planing), splint therapy for temporomandibular dysfunction and bruxism, prosthodontic rehabilitation using custom-fit appliances or implant-supported restorations, and orthodontic decompensation in preparation for orthognathic surgery. In cases of persistent macroglossia or malocclusion, dental teams also contribute to preoperative planning by providing occlusal analysis and appliance-supported stabilization. Importantly, dental follow-up remains essential not only during the active phase of acromegaly, when structural and inflammatory changes progress rapidly, but also in the remission phase. Even after hormonal control is achieved, patients may experience sequelae such as residual malocclusion, temporomandibular dysfunction, mucosal overgrowth, or implant instability. Therefore, dentists play a key role in monitoring late complications, adjusting prosthetic appliances, and maintaining periodontal health in collaboration with endocrinologists and surgeons. These models assume the active involvement of the dentist not only during symptomatic phases but also in preventive care—especially in monitoring therapy-related structural complications such as malocclusion or prosthetic intolerance [12].

A best practice approach implemented in reference centers for acromegaly management involves joint patient consultations carried out by multidisciplinary teams. In the integrated pituitary center model, patients have access to specialists from multiple fields in a single location, enabling coordinated diagnostics and treatment, reducing data fragmentation, and shortening the time to clinical decision-making [58,59]. In more advanced centers, a multidisciplinary team also includes oral and maxillofacial surgeons, dental specialists, ENT consultants, and neuropsychologists. Consultations are typically held every 4 to 6 weeks and involve review of patient imaging, biochemical markers, treatment side effects, and quality of life indicators. Studies from reference institutions report that this structure leads to significantly earlier diagnosis, better hormonal control, and improved dental and prosthetic outcomes. In centers with dedicated dental participation, rates of implant failure and advanced periodontitis were reduced by over 30% compared to general endocrine practice [55]. In such centers, the dentist also acts as a morphological assessment expert, often utilizing imaging (CBCT, cephalometry) and photographic documentation [12].

Recent studies underscore the clinical importance of including dental professionals in the diagnostic and therapeutic management of acromegaly. As Kreitschmann-Andermahr and Kohlmann demonstrated, oro-dental abnormalities such as mandibular prognathism, macroglossia, diastema, and malocclusion are prevalent in patients with active acromegaly and may significantly affect quality of life and prosthetic rehabilitation outcomes. The authors emphasize that systematic dental evaluation should be part of routine monitoring, particularly in those undergoing GH-suppressive therapy or those with planned surgical interventions [60]. Complementary findings by Preo et al. confirmed that dentists and orthodontists often play a pivotal role in early detection. In their cohort of patients with previously undiagnosed acromegaly, characteristic facial and dental changes prompted referral to endocrinologists, accelerating the diagnostic process and reducing the delay in treatment initiation [12]. These studies support the inclusion of dentists as permanent members of multidisciplinary pituitary teams, not only for treatment purposes but also as frontline observers of subtle craniofacial signs suggestive of disease recurrence or progression.

Although numerous guidelines highlight the need for interdisciplinary collaboration in acromegaly care, the integration of dental specialists remains inconsistently implemented across clinical settings. Structural and organizational barriers—such as the absence of routine dental referral protocols and the non-inclusion of oral health assessments in endocrine care pathways—limit the early detection and management of craniofacial complications [55]. As demonstrated by Preo et al., dentists and orthodontists are often among the first to observe characteristic facial features, including mandibular prognathism, diastema, and macroglossia, which may precede the formal diagnosis of acromegaly [12]. Kreitschmann-Andermahr and colleagues also emphasized that oro-dental abnormalities substantially impair prosthetic rehabilitation and quality of life, particularly in patients undergoing somatostatin analog therapy or surgery [60].

Despite this, structural and educational barriers continue to limit the formal integration of dental professionals into pituitary care teams. As highlighted by Coleman et al., incorporating dentistry into interprofessional education and clinical training frameworks may help overcome these silos and promote more consistent collaboration between oral health professionals and medical specialists [61]. These findings suggest that formalizing the role of dentists within multidisciplinary teams—particularly in reference centers—could reduce diagnostic delays and improve long-term functional outcomes.

Modern medicine necessitates a shift away from fragmented specialty-based care toward a structure rooted in data integration, joint clinical decisions, and multi-stage care grounded in communication. In the case of acromegaly—a disease with a complex phenotype and heterogeneous presentation—it is especially critical to ensure continuity between endocrinological and dental care. This can enhance the management of local symptoms and improve overall systemic outcomes. The inclusion of a dentist in the interdisciplinary care team for patients with acromegaly may represent an important but often overlooked component of effective, patient-centered care.

To implement this integration effectively, health systems should consider formal mechanisms for including dental professionals in pituitary MDTs. The proposed formats include quarterly joint consultations, shared access to patient imaging and endocrine reports, and cross-disciplinary education on craniofacial manifestations of endocrine diseases. Institutionalizing referral pathways and embedding dental services within pituitary clinics may reduce diagnostic fragmentation and improve both local and systemic outcomes. Ultimately, a coordinated model grounded in mutual awareness and communication is essential to meet the complex needs of patients with acromegaly.

According to recent international recommendations, the management of acromegaly should be grounded in a fully integrated, multidisciplinary care model. In their 2020 expert consensus, Giustina et al. highlight the necessity of structured collaboration among endocrinologists, neurosurgeons, radiologists, and other key professionals involved in diagnostic and therapeutic processes [55]. Although dental professionals are not explicitly mentioned, their formal inclusion may be particularly relevant in patients with prominent craniofacial or oral manifestations. Such integration could improve the early recognition of symptoms, support the management of treatment-related complications, and enhance long-term functional outcomes.

## 3. Discussion

Craniofacial and oral changes in patients with acromegaly remain one of the most distinct and diagnostically valuable features of this condition, yet their recognition and clinical interpretation continue to be underestimated in endocrinological guidelines and standard care algorithms. Despite the considerable visibility of these features—such as mandibular prognathism, macroglossia, diastemas, and occlusal changes—the potential of dental professionals to participate in the early detection and monitoring of disease activity remains insufficiently utilized in most clinical settings [1].

The persistence of morphological abnormalities in the maxillofacial region even after successful endocrine therapy has been documented in multiple studies. Wagenmakers et al. demonstrated that although long-term remission leads to the stabilization of craniofacial dimensions, bony overgrowth—particularly in the mandible—often persists and does not exhibit significant regression in three-dimensional imaging [18]. This observation has substantial clinical consequences, especially for prosthetic rehabilitation, orthodontic correction, and surgical planning, which must often address irreversible anatomical changes.

A structured summary of common craniofacial manifestations in acromegaly and their estimated reversibility following biochemical remission is presented in Table 4.

Soft tissue manifestations, including macroglossia and gingival hypertrophy, may exhibit a more favorable course in terms of partial regression. Some reports suggest improvement in oral function and prosthetic comfort in patients with biochemical disease control, although high-level comparative data remain scarce [20]. Periodontal health, on the other hand, appears to be influenced by multiple factors—including local hygiene, immune-inflammatory mechanisms, and systemic hormonal status. Patients with acromegaly are frequently affected by periodontitis, with evidence pointing to a potential bidirectional relationship between disease activity and periodontal status [11].

The role of dentists and orthodontists in the diagnostic process has been highlighted in several cross-sectional and retrospective studies. Preo et al. emphasized that dentofacial changes are often first noted by dental professionals, even before an endocrinological diagnosis is made, underlining the need for broader interdisciplinary awareness [12]. However, barriers still remain, including diagnostic delays persist in part due to the fragmentation of care and the absence of clear referral pathways between dental and endocrine services.

Prosthetic and implant planning in patients with active or historical acromegaly poses unique challenges. As reported by Kernen and Bidra, even in the context of disease remission, anatomical alterations and altered bone turnover may affect implant stability and prosthetic fit [45]. While isolated case reports support the feasibility of implant therapy in well-controlled patients, robust longitudinal studies are needed to determine the prognostic impact of endocrine stabilization on implant survival.

In light of these findings, interdisciplinary care models warrant serious consideration. Coordinated management involving endocrinologists, dentists, orthodontists, periodontists, and maxillofacial surgeons has the potential to improve both functional and esthetic outcomes. Nevertheless, the current literature suggests that such collaboration is still implemented inconsistently and primarily limited to academic centers or tertiary referral institutions [6].

There is also a notable lack of structured protocols for long-term dental monitoring in patients with acromegaly. As pointed out by Fleseriu et al. in the Pituitary Society guidelines, dental follow-up is not formally integrated into endocrine treatment algorithms, despite the growing recognition of its relevance to quality of life and functional rehabilitation [15]. Addressing this gap may not only improve oral health but also patient-reported satisfaction and psychosocial well-being.

In conclusion, while the literature confirms the importance of orofacial symptoms in acromegaly—both as diagnostic markers and therapeutic challenges—further research is needed to define optimal standards for interdisciplinary care. Early dental involvement, standardized evaluation protocols, and prospective studies on therapeutic outcomes may collectively enhance the prognosis and quality of life of this patient population.

## 4. Conclusions

1. Changes in the oral cavity and craniofacial structures are among the earliest manifestations of acromegaly and may precede other clinical symptoms; thus, dentists play a key role in early disease detection.
2. Mandibular overgrowth, diastemas, macroglossia, and hypertrophy of the oral soft tissues are characteristic phenotypic features that should prompt diagnostic vigilance in dentists—especially when they progress in adult patients.
3. Active acromegaly negatively affects periodontal health and the oral microbiome, increasing the risk of inflammation, pathological bone remodeling, and biofilm dysregulation.
4. Endocrinological treatment (surgical and pharmacological) results in the partial regression of some oral symptoms but does not reverse established morphological changes; therefore, dental treatment planning should consider the stabilization of biochemical markers.
5. Implant therapy and advanced prosthetic treatment may be effective in acromegalic patients, provided that there is prior assessment of hormonal activity, bone morphology, and periodontal health.
6. Dentists should be permanent members of interdisciplinary teams and participate in treatment planning and monitoring regarding both oral health and craniofacial changes.
7. Integrated care models (e.g., multidisciplinary pituitary centers) offer more effective diagnosis, improved prognosis, and a higher quality of life for patients with acromegaly, and their implementation should be prioritized, including in Central and Eastern Europe.
8. Education on endocrinological diseases, particularly their oral manifestations, should be included in postgraduate and specialty training programs for dental professionals, given the diagnostic role of this professional group.
9. Integrated care for patients with acromegaly must not overlook dentistry, which—based on clinical and epidemiological data—plays a symptomatic and prognostic role, especially in managing complications, enhancing quality of life, and ensuring comprehensive therapy.

## Figures and Tables

**Table 1:** Impact of endocrinological treatment on oral health in acromegaly.

Treatment Modality	GH/IGF-1 Control	Soft Tissue Effects	Skeletal Effects	Dental Implications
Transsphenoidal Surgery	Often achieves biochemical remission (GH suppression and IGF-1 normalization)	Partial regression of soft tissue hypertrophy (tongue, lips, facial muscles)	Irreversible mandibular and maxillary remodeling	Improves prosthetic adaptation; may require orthognathic procedures
Somatostatin Analogs (SSAs)	Reduces both GH and IGF-1 levels	Reduces gingival overgrowth and periodontal inflammation	No reversal of bone changes	Enhances periodontal health; facilitates prosthetic planning
Dopamine Agonists	Limited GH/IGF-1 suppression	Minor or unclear effect on soft tissues	No documented skeletal effects	Adjunctive role; limited dental impact
Pegvisomant	Normalizes IGF-1 without GH suppression	Improves prosthetic comfort; reduces soft tissue bulk	No effect on bone morphology	Beneficial for articulation and mastication; monitor for persistent skeletal discrepancies
Pituitary Radiotherapy	Delayed hormonal control; risk of hypopituitarism	Indirect effects via hormonal deficiencies (possible tissue atrophy)	Decreased bone mineral density; potential alveolar bone fragility	Implant planning requires BMD evaluation and hormonal stabilization
Combined Therapy	Greater likelihood of long-term disease control	Stabilization of soft tissue state with early treatment	Persistent osseous deformities, especially if diagnosed late	Requires long-term interdisciplinary coordination; adjust timing of dental procedures

**Table 2:** Clinical considerations and recommendations for implant and prosthetic treatment in acromegaly.

Clinical Factor	Pathophysiological Implication	Impact on Dental Treatment	Management Strategy
Active acromegaly (↑ GH/IGF-1)	Impaired bone remodeling, increased metabolic turnover	Higher risk of implant failure, poor healing	Delay elective procedures until hormonal control is achieved
Mandibular prognathism/maxillary narrowing	Occlusal disharmony, loss of centric occlusion	Reduced prosthetic stability; difficulty with denture retention	CBCT-guided occlusal planning; consider orthognathic evaluation
Soft tissue hypertrophy (e.g., macroglossia, gingival overgrowth)	Altered mucosal dynamics and soft tissue pressure	Discomfort with removable prostheses; peri-implant soft tissue complications	Use of implant-supported fixed prostheses; individual tissue management
Periodontal disease and tooth mobility	Reduced interarch stability; risk of peri-implantitis	Poor prognosis for conventional prosthodontics	Comprehensive periodontal treatment; consider splinting or selective extractions
Metabolic comorbidities (e.g., diabetes, hypovitaminosis D)	Altered wound healing and immune response	Increased risk of complications and delayed osseointegration	Optimize systemic health: glycemic control, vitamin D/Ca supplementation
Long-standing skeletal changes	Irreversible craniofacial remodeling	Prosthetic instability, need for custom alignment	Consider patient-specific implants (PSI), 3D surgical planning
History of uncontrolled acromegaly	Persistently altered bone microarchitecture	Marginal bone loss around implants	Close radiological monitoring; avoid early loading protocols

**Table 3:** Determinants of periodontal disease and microbial imbalance in acromegaly.

Factor	Mechanism	Clinical Consequences	Management Recommendation
Excess GH/IGF-1	↑ IL-1β, IL-6, TNF-α; prolonged neutrophil activation	Gingival hypertrophy, inflammation, connective tissue degradation	Systemic hormonal control; integration with local periodontal therapy
Salivary dysfunction	↓ Peroxidase, ↓ α-amylase, ↓ bicarbonate → impaired buffering and antimicrobial defense	Oral dysbiosis, increased plaque retention, reduced resistance to pathogens	Use of oral probiotics; avoid alcohol-based rinses; consider salivary diagnostics
Macroglossia and arch hypertrophy	Anatomic crowding and malposition of teeth	Mechanical difficulty with brushing, plaque accumulation	Modified interdental brushes, water flossers; individualized oral hygiene instruction
TMJ dysfunction and limited jaw opening	Structural changes: condylar thickening, disk displacement	Reduced access to posterior teeth; hygiene difficulties; masticatory discomfort	Frequent professional cleanings; soft brush techniques; pain management if needed
Persistent inflammation despite biochemical remission	Systemic-local feedback via cytokines (esp. IL-6, and TNF-α)	Variable treatment response; ongoing periodontal risk	Periodontal recall every 3–4 months; interdisciplinary monitoring
Somatostatin analogs (SSAs)	May reduce salivary flow; indirect anti-inflammatory effects	Subclinical xerostomia; reduced mucosal protection	Moisturizing rinses; monitor mucosal status; reinforce mechanical plaque control

**Table 4:** Common craniofacial and oral manifestations of acromegaly, with assessment of reversibility and clinical implications.

Type of Change	Anatomical Location	Tissue Type	Reversibility After Remission	Clinical Relevance
Macroglossia	Tongue	Soft tissue	Partial to full	Affects articulation, breathing, and denture stability
Gingival hypertrophy	Gingiva	Soft tissue	Partial	Impairs hygiene, causes bleeding and discomfort
Diastemas	Anterior maxilla/mandible	Bone/teeth	Low	Requires orthodontic management
Mandibular prognathism	Mandible	Bone	Very low	May require orthognathic surgery
Alveolar bone hypertrophy	Maxilla and mandible	Bone	Low	Affects implant planning and prosthetic design
TMJ dysfunction	Temporomandibular joint	Bone/cartilage	Variable	Associated with occlusal instability and pain

## Data Availability

No new data were created or analyzed in this study. Data sharing is not applicable to this article.
